# Women in Microsurgery

**DOI:** 10.1055/a-2737-7475

**Published:** 2026-02-27

**Authors:** Gemma Pons

**Affiliations:** 1 Department of Plastic and Reconstructive Surgery, Hospital de la Santa Creu i Sant Pau, Barcelona, Catalunya, Spain

**Figure:**
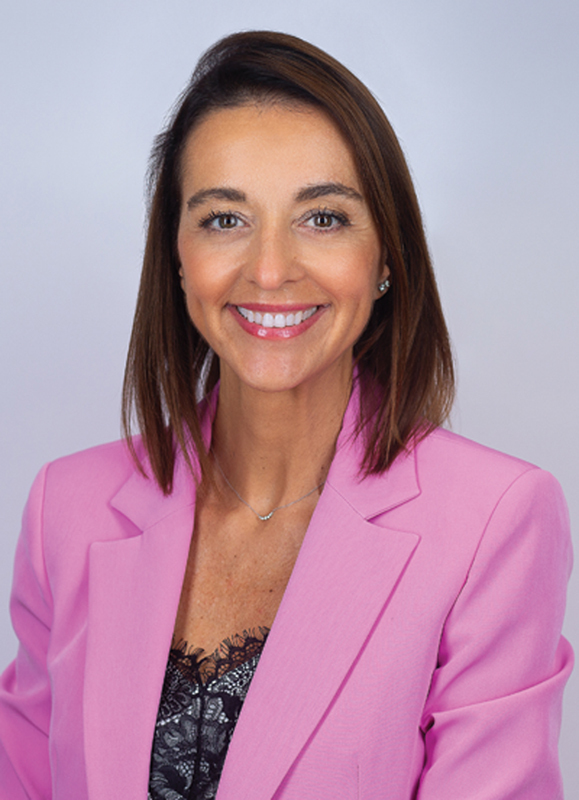
Gemma Pons, MD, PhD

According to Hong et al's article, 1 the attributes of a good microsurgeon include clarity, curiosity, perseverance, passion, open-mindedness, and action—qualities equally represented in both men and women. Microsurgery is a technically demanding and innovative discipline that captivates those who truly feel passion for it. It is precisely this passion that gives microsurgeons, regardless of gender, the strength and balance to overcome all possible challenges.

In recent years, an increasing number of women have entered plastic surgery and microsurgical subspecialties. Their perseverance, vision, and leadership are steadily reshaping the paradigm of women's roles in microsurgery. However, historically, women have been underrepresented in the field. Dr. Alma Dea Morani, admitted to the American Society of Plastic and Reconstructive Surgeons in 1948, was the first recognized woman plastic surgeon. Despite limited mentorship and institutional support, her determination and perseverance enabled her to build a successful career, becoming a role model for future generations. 2

Thanks to the courage and resilience of pioneering women, we are now witnessing a global transition in which women are increasingly represented and influential across all areas of our specialty. Nevertheless, female representation in academic and leadership positions remains low, and barriers to advancement and retention persist, particularly in low-income countries. At the same time, for most of human history, leadership has been dominated by men, and achieving a balanced and inclusive professional world inevitably requires time and collective effort. We can, however, look toward an optimistic horizon, where determination, passion, and dedication will place women in the positions they deserve.

Although studies have shown no significant gender-based differences in microsurgical skills, social and cultural factors may still hinder women from pursuing their professional goals. In this context, mentorship plays a crucial role in providing guidance, support, and encouragement. I have been fortunate to have an active and generous male mentor who has profoundly influenced my career. He has invested time and energy in understanding my strengths, weaknesses, and aspirations, celebrating each achievement, and continuously encouraging me to pursue my dreams. Along my path, I have also met extraordinary microsurgeons, both men and women, who became mentors unintentionally, serving as role models who have inspired me professionally and personally, and, most importantly, have become dear friends.

However, mentorship alone is not enough if one cannot recognize and seize the opportunities that foster growth. Patience, hard work, dedication, and honesty are essential to building a strong career in microsurgery. These values are universal, shared by both men and women, until the natural desire for motherhood arises. This is often a critical moment in a female microsurgeon's career, one that can define her professional trajectory. As Claudia Goldin, Nobel Laureate in Economic Sciences (2023), 3 demonstrated, gender gaps in income and advancement often emerge when women have children, as they temporarily or permanently reduce their workload, limiting both earnings and professional growth. In my opinion, motherhood is an incomparable achievement in a woman's life, and it should not represent an obstacle to maintaining passion and commitment to one's profession. Nonetheless, even today, many cultural and social pressures often make female microsurgeons feel guilty when trying to balance motherhood and career. I believe, however, that when children grow up seeing a mother who works with joy and passion, they grow up healthy, and the mother becomes a role model for them. Ultimately, what matters most is the quality of time shared, rather than its quantity. But work still has to be done, by women themselves and by institutions, to make this work–life balance a true reality.

Overall, the journey toward equality in microsurgery is an ongoing process. It still requires collective recognition, institutional support, and mentorship that transcends gender. Nevertheless, with determination, empathy, and passion—the same values that define exceptional microsurgeons—our specialty can continue to grow through talent, diversity, and collaboration.
